# Molecular mechanisms linking stress and insulin resistance

**DOI:** 10.17179/excli2021-4382

**Published:** 2022-01-24

**Authors:** Habib Yaribeygi, Mina Maleki, Alexandra E. Butler, Tannaz Jamialahmadi, Amirhossein Sahebkar

**Affiliations:** 1 Research Center of Physiology, Semnan University of Medical Sciences, Semnan, Iran; 2 Urology and Nephrology Research Center, Shahid Beheshti University of Medical Sciences, Tehran, Iran; 3 Research Department, Royal College of Surgeons in Ireland, Bahrain, PO Box 15503, Adliya, Bahrain; 4 Department of Nutrition, Faculty of Medicine, Mashhad University of Medical Sciences, Mashhad, Iran; 5 Applied Biomedical Research Center, Mashhad University of Medical Sciences, Mashhad, Iran; 6 Biotechnology Research Center, Pharmaceutical Technology Institute, Mashhad University of Medical Sciences, Mashhad, Iran; 7 School of Medicine, The University of Western Australia, Perth, Australia; 8 Department of Medical Biotechnology and Nanotechnology, Faculty of Medicine, Mashhad University of Medical Sciences, Mashhad, Iran; 9 Department of Biotechnology, School of Pharmacy, Mashhad University of Medical Sciences, Mashhad, Iran

**Keywords:** diabetes mellitus, insulin resistance, stress, immune system, insulin signal transduction

## Abstract

To date, there is ample evidence to support the strong relationship between stress and insulin resistance. While diabetes mellitus acts as a potent stress inducer, stress may be an upstream event for insulin resistance as well. It is widely recognized that diabetes mellitus is more prevalent among people who have a stressful lifestyle; however, the underlying mechanisms are not well understood. In the current study, we surveyed the scientific literature for possible interactions between stress and insulin resistance and found that stress can impair glucose homeostasis, working through at least six molecular pathways.

## Introduction

The global incidence of diabetes mellitus (DM) is growing rapidly (Andes et al., 2019[6]). This chronic disorder adversely affects most metabolic processes and results in diabetes-induced complications and tissue dysfunction (Forbes and Cooper, 2013[34]). DM underlies many disabilities and/or deaths in humans and presents a major challenge for healthcare provider systems throughout the world (ADA, 2018[2]; Sussman et al., 2020[125]). Therefore, many studies have focused upon developing new preventive and/or therapeutic protocols (Kusminski et al., 2016[64], Wong et al., 2017[137], Yaribeygi et al., 2018[141]). Although the exact pathophysiology of DM is not clearly established, some associated risk factors which increase the risk of insulin resistance and DM are well recognized (Kusminski et al., 2016[64]; Zaccardi et al., 2016[145]; Yaribeygi et al., 2021[140]). 

Stress is an inevitable part of everyday life and is defined as any state that imposes undesirable conditions on the subjects (Hatef et al., 2016[44]; Patch and Figueredo, 2017[96]). Many local and systemic physiological pathways, such as neural networks and hormonal systems, are stimulated under stressful situations. Stress may therefore have complicated interactions with a myriad of pathophysiologic states including insulin resistance and DM (Wellen and Hotamisligil, 2005[135]; Yaribeygi et al., 2017[142]; Yaribeygi and Sahraei, 2018[143]). Stress is a very broad term, encompassing allostasis, allostatic load, psychological and emotional burdens, physical forces and biochemical changes (Keay and Bandler, 2001[59]; McEwen, 2000[85]). In the current review, we focus our discussion upon *chronic emotional and psychological stresses,* which are now recognized to be threats to human health, especially in industrial communities. These types of stresses may be induced by many disparate stimuli, such as chronic load noise, air pollution, divorce, dismissal from work, missing child or parents, among others. Although it is well known that minimal or acute stress might be essential in certain circumstances to improve bodily function by working to accelerate nervous processes and enhancing survival (Yaribeygi et al., 2017[142]), strong associations have linked chronic psychological stress with disorders such as cardiovascular disease and metabolic dysfunction (Yaribeygi et al., 2017[142]). In this context, it has been suggested that stress could increase the risk of insulin resistance and lead to a higher incidence of DM (Surwit et al., 1992[124]; Wellen and Hotamisligil, 2005[135]; Li et al., 2013[68]). Since association between stress and insulin resistance has not been extensively studied, the aim of the current review was to present the possible molecular interactions by which stress can impair insulin signaling and induce insulin resistance. 

### Physiology of stress and stress response 

Stress is a common term referred to as any stimulus imposing undesirable conditions on the subject (Yaribeygi and Sahraei, 2018[143]). This can also be described as any external threat emanating from various sources, including physical, chemical, biological, and psychological agents (known as stressors), which tend to disturb the physiological homeostasis of the body (Yaribeygi and Sahraei, 2018[143]). These stressors commonly induce a reaction, known as the stress response, to regain the physiologic balance of the organism and increase its survival (Yaribeygi and Sahraei, 2018[143]). The stress response is mediated via three major pathways: the hypothalamus-pituitary-adrenal (HPA) axis, the renin-angiotensin system (RAS) and the automatic nervous system (ANS). These pathways are further subclassified into the rapid phase (autonomic nervous system) and the slow phase (HPA axis) of the stress response (Yaribeygi et al., 2017[142]; Yaribeygi and Sahraei, 2018[143]). However, these compensatory responses may further exacerbate any pre-existing condition and act as new threats to the body's homeostasis (Yaribeygi et al., 2017[142]; Yaribeygi and Sahraei, 2018[143]).

The HPA axis is comprised of three endocrine organs, the hypothalamus, pituitary and adrenal glands (Tsigos and Chrousos, 2002[128]; McCormick et al., 2010[82]). The hypothalamus directs adrenocorticotropic hormone (ACTH) release from the anterior pituitary by releasing Corticotropin Releasing Hormone (CRH, or CRF). Arginine vasopressin (AVP or ADH) is also a pivotal hormone, functioning with synergistic effect on the ACTH response (Tsigos and Chrousos, 2002[128]; Carrasco and Van de Kar, 2003[17]; Herman et al., 2003[46]). CRH is subsequently transported via portal vessels to the pituitary, where it binds to specific receptors and initiates the synthesis of a polypeptide known as Pro-opiomelanocortin (POMC), which is the precursor for smaller peptides such as beta endorphins, ACTH and MSH (Sawchenko, 1987[110]; Herman et al., 2003[46]). ACTH then moves via the circulation to the cortical layer of the adrenal glands and, thereby, induces the biosynthesis and release of two forms of adrenocorticosteroids: glucocorticoids and mineralocorticoids (Cullinan et al., 1995[23]). Glucocorticoids are the final effectors of the HPA axis that are directly involved in the stress response, as well as maintaining the basal level of HPA activity and terminating the stress responses through a negative feedback mechanism (Cullinan et al., 1995[23]; Carrasco and Van de Kar, 2003[17]).

The renin-angiotensin system (RAS), which is primarily involved in regulating body fluids and electrolytes, is also involved in the stress response (Boron and Boulpaep, 2012[12]). This system is highly sensitive to changes in plasma concentration, blood pressure and blood volume, and reacts rapidly by releasing angiotensin II (Khanna et al., 2017[60]). Angiotensin II primarily acts as a very potent vasoconstrictor (Khanna et al., 2017[60]). It also induces the release of aldosterone from the adrenal glands and mediates salt homeostasis (mainly sodium and potassium) (McCormick and Bradshaw, 2006[83]). Renin triggers the conversion of angiotensinogen into angiotensin I which then forms angiotensin II in pulmonary epithelial cells. (McCormick and Bradshaw, 2006[83]). Since receptors of angiotensin II (type 1 (AT1) and type 2 (AT2)) are widely distributed in the brain, RAS activation is closely involved in fast elements of stress responses (Carrasco and Van de Kar, 2003[17]; Saavedra et al., 2005[108]).

The autonomic nervous system (ANS) is another system involved in the stress response (Yaribeygi and Sahraei, 2018[143]). This highly reactive network reacts to any stimuli disturbing body fluids, blood pressure or cardiovascular activities through either the sympathetic or parasympathetic nervous system, resulting in chronotropic and/or inotropic effects (Corr et al., 1986[22]). Activation of the ANS also has numerous metabolic outcomes leading to mobilization of substrates (Cacioppo et al., 1998[15]). While the sympathetic system mainly acts via releasing catecholamines from the adrenal glands, the parasympathetic nervous system mostly acts by activation of the ambiguous nucleus and dorsal motor nucleus of vagal nuclei (Kandel et al., 2000[57]) (Table 1(Tab. 1)). 

Other biological systems, such as the locus-ceruleus/norepinephrine (LC/NE) system, the endogenous opioid system (EOS), dopamine and the mesocorticolimbic system and the glutamate system are also involved in the stress response (Yaribeygi and Sahraei, 2018[143]). All of these physiologic systems regulate the metabolic aspects of the stress response such as glucose homeostasis (Yaribeygi et al., 2017[142]; Yaribeygi and Sahraei, 2018[143]). 

## Insulin Resistance and Diabetes Mellitus

**Figure 1:**
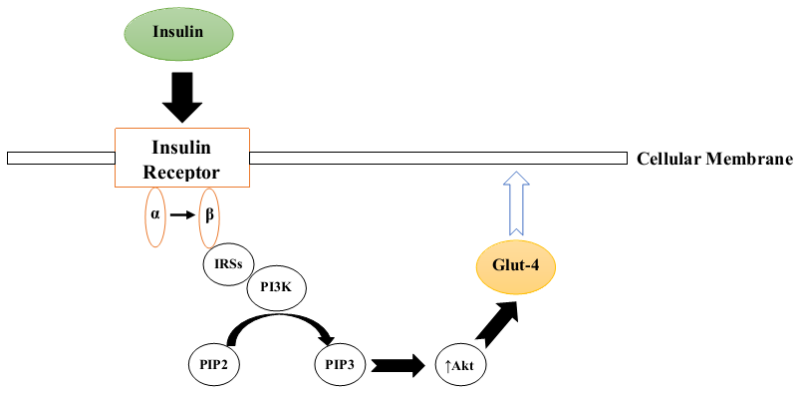
Simplified schematic representation of the insulin signal transduction pathway (IRSs= insulin receptor substrates, PI3K= Phosphoinositide 3-kinase, PIP2= Phosphatidylinositol 4,5-bisphosphate, PIP3= Phosphatidylinositol 3,4,5-trisphosphate, Akt= protein kinase B, Glut-4= glucose transporter type 4)

Insulin is a metabolic hormone released exclusively by pancreatic beta cells (Petersen and Shulman, 2018[98]). This polypeptide has significant effects on most metabolic pathways including lipids, carbohydrates and proteins and, hence, its normal signaling/function is critical for maintaining homeostasis in the body (Petersen and Shulman, 2018[98]). Insulin acts via a series of sequential complex steps known as the insulin signal transduction (IST) pathway which is precipitated by the binding of insulin to the α chain of its specific receptor, known as the insulin receptor (IR) (Færch et al., 2016[33]). This binding induces structural changes in the receptor β chain followed by recruitment of different adaptor proteins, insulin receptor substrates (IRSs), Shc protein (SHC-transforming) and APS protein (adapter protein with a PH and SH2 domain) which, in turn, provide appropriate binding sites for IRS-1 (Kiselyov et al., 2009[62]; Hall, 2015[43]). Activated IRS-1 binds to PI3K (phosphoinositide 3-kinase) which promotes the conversion of phosphatidylinositol 4,5-bisphosphate (PIP_2_) to phosphatidylinositol 3,4,5-trisphosphate (PIP_3_) (Ho et al., 2016[48]). PIP_3 _is a potent inducer of protein kinase B (PKB, also known as Akt), which facilitates glucose entry into the cells by translocalization of glucose transporter type 4 (GLUT-4) to the cell membrane (Figure 1(Fig. 1)) (Ho et al., 2016[48]; Koeppen and Stanton, 2017[63]). 

There are numerous types of kinases such as ERK1/2 (extracellular signal‐regulated kinase 1/2), atypical PKC (protein kinase C), S6K1 (ribosomal protein S6 kinase beta-1), SIK2 (serine/threonine-protein kinase 2), mTOR (mammalian target of rapamycin), ROCK1 (rho-associated protein kinase-1), AMPK (AMP-activated protein kinase) and GSK3 (glycogen synthase kinase) which are also able to phosphorylate IRSs and facilitate insulin signal transduction (Kiselyov et al., 2009[62]; Copps and White, 2012[21]; Petersen and Shulman, 2018[98]).

## Classification of Diabetes Mellitus

DM is commonly categorized into three main classes as type1, type2 and gestational diabetes (ADA, 2017[3]). Type1 DM (T1DM) or insulin-dependent diabetes mellitus (IDDM) mainly refers to a state where insulin levels are extremely low due to autoimmune targeting and destruction of beta cells (ADA, 2017[3]). Type2 DM (T2DM) or non-insulin-dependent diabetes mellitus (NIDDM), the most prevalent form of DM, results from a combination of insulin resistance in peripheral tissues together with beta cell loss through apoptosis (ADA, 2017[3]). Gestational diabetes is another form of DM that, by definition, develops during pregnancy probably due to hormonal disturbances (de Faria Maraschin, 2012[25]). There are also less common forms of DM such as Latent Autoimmune Diabetes in Adults (LADA), Maturity-Onset Diabetes of the Young (MODY) and diabetes secondary to medical conditions (such as pancreatitis) or medications (such as corticosteroids) (ADA, 2014[1]; O'Neal et al., 2016[91]). T2DM is by far the most common form of DM and is closely linked to dysfunction in IST and insulin resistance (ADA, 2017[3]). 

## Stress and Diabetes Mellitus

Stress and DM have mutual interactions, meaning that both stress and DM can induce or intensify one another (Lloyd et al., 2005[75]). Eating restrictions and therapeutic medications are considered as stressors for diabetic patients (Lloyd et al., 2005[75]; Tsenkova et al., 2013[127]; Martyn-Nemeth et al., 2014[81]). It has been well documented that diabetic patients have higher levels of anxiety, depression, anger and stress-related behavioral disorders than the non-diabetic population (Anderson et al., 2001[5]; Grigsby et al., 2002[40]; Whittemore et al., 2004[136]; Jones et al., 2016[55]). On the other hand, people who are chronically stressed have poor glycemic control to varying degrees (Yang et al., 2016[138]). The evidence indicates that stressful life events, traumatic experiences, general emotional stress, anger and hostility, distressed sleep and workplace stress may negatively modulate glucose homeostasis and induce insulin resistance (Lustman et al., 2000[76]; Lin et al., 2004[72]; Schneiderman et al., 2005[111]; Alexander et al., 2007[4]; Salleh, 2008[109]). Therefore, these stressors are now considered to be independent risk factors for DM (Grigsby et al., 2002[40]; Lin et al., 2004[72]; Razzoli et al., 2017[103]; Yaribeygi et al., 2020[144]) and individuals with higher levels of chronic stress are more likely to develop DM when compared to their non-stressed counterparts (Razzoli et al., 2017[103]). However, the inter-relationships governing this association are poorly understood. 

Studies also demonstrate a relationship between stress-derived behavioral changes and development of diabetes. Stress can negatively impact lifestyle, particularly in those people with a lower capacity to cope with the imposed stress (Lloyd et al., 2005[75]). For example, overfeeding and hyperphagia, which can severely disrupt metabolic processes and gradually induce insulin resistance, are common amongst stressed individuals (Wardle et al., 2000[133]; Lloyd et al., 2005[75]; Razzoli et al., 2017[103]). Stressed individuals usually consume high calorie food as a coping mechanism to withstand their stressful conditions (Lloyd et al., 2005[75]; Scott et al., 2012[112]). In a study conducted by Chandola et al., in 2006, a chronic work stress environment was positively correlated with insulin resistance in British civil servants (Chandola et al., 2006[18]). In addition, levels of physical activity are lower and a sedentary lifestyle more prevalent amongst stressed subjects when compared with the non-stressed population (de Sousa et al., 2014[26]; Stults-Kolehmainen and Sinha, 2014[120]; Panahi and Tremblay, 2018[92]). Thus, stress-induced unhealthy lifestyle accounts for many of the potential risk factors for poor health and markedly increase the risk of metabolic disturbances and insulin resistance (de Sousa et al., 2014[26]). 

Therefore, stress-related behavioral changes, along with hormonal and neuronal interactions, may be key factors connecting stress and insulin resistance (Lloyd et al., 2005[75]). It is well recognized that stress hormones, such as cortisol, epinephrine, growth hormone and glucagon, induce acute transient hyperglycemia that occurs in a "fight or flight" reaction (Shiloah et al., 2003[114]; Goldstein, 2010[38]). However, in the following sections, we only focus on the steady state negative effects of stress and the stress response on glucose homeostasis, insulin sensitivity and DM.

### 1. Stress and β-cell function 

A healthy, well-functioning beta cell (β-cell) mass is an absolute requirement for normal glucose homeostasis, since these specialized cells alone produce and secrete insulin (Gerber and Rutter, 2017[37]). β-cells represent a small proportion of the total pancreatic tissue mass (≤ 1 %) (Butler et al., 2010[14]). Therefore, any disruption to their normal functioning can significantly and negatively impact glucose homeostasis (Gerber and Rutter, 2017[37]). There are growing numbers of "stress markers" identified that relate to the level of beta cell function, especially in T1DM (Wali et al., 2015[131]; Mirmira et al., 2016[87]). Shiloah et al., in 2003 reported that acute psychological stress promotes beta cell death in non-diabetic patients. They found that beta cell function and glucose homeostasis are inversely associated with psychological stress (Shiloah et al., 2003[114]). Recently, in 2019, Bebbington et al., demonstrated that stress and anxiety have a deleterious impact on beta cell function, leading to blood glucose fluctuations in TIDM patients. They determined that the emotional state of these patients has a significant impact on insulin release and is able to alter glucose homeostasis (Bebbington et al., 2019[9]). In 2014, Parkulo reported that chronic stress exerted negative effects on the morphology of pancreatic islets and reduced the functional mass of pancreatic islet cells in mice, leading to islet atrophy and onset of TIDM; further, chronic stress down-regulated genes involved in beta cell proliferation (Parkulo, 2014[95]). Huffman et al., in 2013 conducted a study examining the potential relationship between depression and beta cell function, finding that some patients with depression and psychological stress had lower beta cell efficiency as well as reduced insulin sensitivity (Huffman et al., 2013[51]). Taken together, these data strongly suggest that stress has a negative impact on glucose homeostasis by modifying beta cell function (Shiloah et al., 2003[114]; Huffman et al., 2013[51]; Parkulo, 2014[95]; Bebbington et al., 2019[9]). These adverse effects may be due to overstimulation of the immune response which, in turn, promote higher levels of inflammatory mediators, and the recruitment of inflammatory cytokines and/or of pro-apoptotic agents into the pancreatic islets (Hackett and Steptoe, 2017[42]).

### 2. Stress and lipid metabolism

Dyslipidemia, a common feature of diabetes, has been shown to markedly reduce insulin sensitivity over time due to the negative effects on IST (Howard, 1999[49]; Athyros et al., 2018[8]). Therefore, lipid profile improvement is a key therapeutic target in diabetic patients (Dyson et al., 2018[28]). Stress triggers a cascade of neurohormonal responses, including ANS, RAS, glucagon and glucocorticoids, which are all involved in the regulation of lipid metabolism (Rodríguez-Sureda et al., 2007[105]). Based on experimental studies, stress modifies most enzymatic functions involved in lipid metabolism, such as hepatic lipase, lipoprotein lipase, cholesterol esterase, HMG-CoA reductase, acyltransferase, acyl-CoA dehydrogenase, enoyl-CoA hydratase, 3-hydroxyacyl-CoA dehydrogenase, and 3-ketoacyl-CoA thiolase which is mainly expressed in the liver and adipose tissue (Rodríguez-Sureda et al., 2007[105]; Suozzi et al., 2009[122]; Chuang et al., 2010[20]; Parekh et al., 2017[94]). Chuang et al., in 2010 demonstrated that social stress downregulated genes involved in lipid metabolism. They reported that imposed stress reduced the transcription of liver X receptor (LXR), sterol regulatory element-binding protein 1 (SREBP1c) and carbohydrate-responsive element-binding protein (ChREBP) and led to impaired lipid metabolism in hepatic cells of animals (Chuang et al., 2010[20]). Also, Suozzi and colleagues in 2009 found that stress profoundly changed the distribution of fatty acid synthase and long-chain fatty acyl-CoA synthetase in fatty acid synthesis and degradation, respectively. They demonstrated that stress seriously modulates lipid metabolism not only via regulating gene expression but also by affecting distribution of essential enzymatic elements in liver and adipocyte tissues (Suozzi et al., 2009[122]). Lieberman et al., in 2012 reported that dysregulated lipid metabolism was closely related to depressed mood (Lieberman et al., 2012[70]). Recently, Mellon and coworkers in 2019 found similar results and reported that patients with PTSD (post-traumatic stress disorder) have impaired lipid metabolism (Mellon et al., 2019[86]). Collectively, this data suggests that stress has negative downstream effects on lipid metabolism and may help to explain the linkage between stress and insulin resistance. These effects may be exerted at the gene level or via neurohormonal systems involved in stress response such as ANS, RAS or stress hormones.

### 3. Stress, the immune system and inflammation 

Inflammatory responses have pivotal roles in the pathophysiology of insulin resistance and DM (Shoelson et al., 2006[116]). Based on previous studies, inflammation negatively regulates the insulin signaling pathways and increases the risk of DM (Tilg and Moschen, 2008[126]; Saad et al., 2016[106]; Shimobayashi et al., 2018[115]; Yaribeygi et al., 2019[139]). Inflammatory mediators, including numerous proinflammatory cytokines, are activated in response to stimuli such as infection, injury, trauma and stress (Shoelson et al., 2006[116]; Chovatiya and Medzhitov, 2014[19]). Chronic hyperglycemia is an example of a homeostatic imbalance which stimulates the stress response as well as inflammatory events (Chovatiya and Medzhitov, 2014[19]).

Inflammatory responses are markedly increased in stress-related diseases, meaning that stress can indirectly trigger inflammation (Chovatiya and Medzhitov, 2014[19]). Black et al., in 2002 reported that psychosocial stress could stimulate inflammatory responses involved in cardiovascular disorders. They stated that inflammatory responses are a component of the stress response, which evolved later, and were adaptive in that an animal was better able to react to an invading organism or agent. Stress has also been shown to induce recruitment of inflammatory cells through activating a number of neurohormonal systems and thereby promote inflammatory processes in the target tissues. Moreover, the release of corticosteroids and catecholamines in response to HPA activation induced the recruitment of lymphocytes and monocytes which, in turn, aggravated the systemic inflammatory events as well as inflammation in pancreatic beta cells (Black and Garbutt, 2002[11]). 

Similarly, inflammation can promote the stress response, as there is mutual interaction between these systems (Black and Garbutt, 2002[11]; Kiecolt-Glaser et al., 2010[61]). Some proinflammatory mediators such as LPS (lipopolysaccharide) on gram negative bacteria can activate the HPA axis and provoke a stress response (Walker et al., 2009[132]; Lin et al., 2012[73]). Inflammatory mediators, such as tumor necrosis factor alpha (TNF-α), can induce stress-related behaviors in experimental models (Kaster et al., 2012[58]). By contrast, relieving stress can attenuate inflammatory responses via action upon central pathways and reducing norepinephrine (NE) and/or other catecholamine release (Black and Garbutt, 2002[11];Kiecolt-Glaser et al., 2010[61]; Chovatiya and Medzhitov, 2014[19]). It is well established that central catecholamines are involved in both stress and inflammation. NE is able to provoke peripheral inflammatory responses when released from stimulated sympathetic neurons (Dulaney et al., 1992[27]; Black and Garbutt, 2002[11]). Tissue-resident macrophages are strongly influenced by NE and/or other catecholamines released in response to stress (Venkatachalam and Montell, 2007[130]). Thus, HPA axis activation can virtually translate into an inflammatory process which is mediated via extra/intra cellular signals (Julius and Basbaum, 2001[56]; Black and Garbutt, 2002[11]; Venkatachalam and Montell, 2007[130]; Qian et al., 2011[102]; Chovatiya and Medzhitov, 2014[19]). Clinical evidence has indicated that inflammation of varying degrees, together with insulin resistance, is present in individuals exposed to chronic stress (Postal et al., 2016[100]; Lyra e Silva et al., 2019[77]). Postal et al., in 2016 demonstrated high levels of TNF-α, a major inflammatory marker, in patients with insulin resistance and depression (Postal et al., 2016[100]). Johnson and colleagues in 2017 provided further evidence indicating that memory loss and depressive symptoms are correlated with inflammation and insulin resistance (Johnson et al., 2017[53]). Likewise, Himmerich and coworkers in a clinical study in 2008 found that a depressive disorder contributed to the inflammatory response and insulin resistance (Himmerich et al., 2008[47]). These findings strongly imply that stress can provoke insulin resistance, at least in part, by promoting inflammatory responses.

### 4. Stress and the autonomic nervous system 

The ANS is a substantial neuronal network regulating many involuntary, spontaneous and high-level physiological functions in humans (McCorry, 2007[84]). This system consists of central and peripheral neuronal structures that are widely distributed in most tissues and can thereby exert major effects on body homeostasis as well as peripheral insulin sensitivity (Esler et al., 2001[32]; Lindmark et al., 2003[74]; McCorry, 2007[84]). The ANS has two main components, the sympathetic and parasympathetic nervous systems (Guarino et al., 2017[41]). While the sympathetic nervous system affects target tissues via epinephrine or NE, the parasympathetic nervous system directs its regulatory functions via release of acetylcholine (Guarino et al., 2017[41]). 

The ANS plays a major role in energy and glucose metabolism, since it has potent regulatory effects on metabolic hormones such as cholecystokinin, neuropeptide YY, pancreatic polypeptide, glucagon like peptide-1, ghrelin, insulin and leptin (Guarino et al., 2017[41]). The ANS also plays a significant role in energy expenditure and lipolysis which both indirectly modulate insulin sensitivity in the long term (Turner et al., 2014[129]; Guarino et al., 2017[41]). The afferent vagal nerve also has significant interactions with the aforementioned metabolic hormones (Guarino et al., 2017[41]). Sympathetic innervations are closely involved in chronic weight loss and energy expenditure through mobilization of white and brown adipocytes which, in turn, affect insulin sensitivity (Guarino et al., 2017[41]). Thus, it is now well established that the ANS has modulatory effects on insulin sensitivity and that the level of its activity contributes to the pathophysiology of insulin resistance and DM (Surwit and Feinglos, 1988[123]; Esler et al., 2001[32]; Johnson et al., 2012[54]; Ekambaram et al., 2013[30]; Turner et al., 2014[129]). 

Previous clinical studies have documented the role of the ANS in regulation of glucose homeostasis and insulin sensitivity (Pardo et al., 2007[93]). Pardo et al., in 2007, in a study on patients with depressive disorders, found that vagal nerve stimulation improved glucose homeostasis and induced weight loss in these patients (Pardo et al., 2007[93]). Huang and coworkers in 2014 provided further evidence, suggesting that parasympathetic stimulation improved glucose tolerance and increased insulin sensitivity in prediabetic patients (Huang et al., 2014[50]). Camilleri et al., in 2008 reported that intermittent vagal nerve blocking is correlated with improved glucose homeostasis and increased energy expenditure (Camilleri et al., 2008[16]). Furthermore, Shikora and colleagues in 2013 showed that the parasympathetic nervous system is involved in insulin sensitivity and glucose homeostasis in patients with T2DM (Shikora et al., 2013[113]).

It has previously been reported that higher levels of fasting plasma insulin (a marker of insulin resistance), are closely related to the low-to-high frequency (LF/HF) ratio of heart rate variability (an index of the sympatho-vagal balance) (Emdin et al., 2001[31]). Epidemiological studies have also found a correlation between insulin resistance and sympatho-vagal balance (Modan and Halkin, 1991[88]; Skyler et al., 1995[118]). In diabetic patients, hypertension is more prevalent than in non-diabetic individuals (Moreira et al., 2015[89]). However, insulin sensitivity is lower in hypertensive patients and thereby, it is fair to assume that insulin resistance and hypertension, as in the metabolic syndrome, are associated with sympathetic nervous system overactivation (Frontoni et al., 2005[35]). Correspondingly, sympathetic deactivation has been shown to improve insulin sensitivity in patients with metabolic syndrome (Mancia et al., 2006[80]). These results confirm the relationship between ANS activity and insulin sensitivity. 

The autonomic stress response is commonly mediated via ANS activity (Yaribeygi and Sahraei, 2018[143]). Moreover, chronic stress is a potential contributor to the development of DM and enhanced stress-induced sympathetic activation has been related to insulin resistance (Mancia et al., 2007[80]; Moreira et al., 2015[89]). Hence, modulating ANS activity is a favorable therapeutic approach to improve glucose homeostasis (Anichkov et al., 2005[7]). Prolonged exposure to psychological stress can overstimulate the sympathetic nervous system and result in excessive levels of circulating catecholamines which, in turn, impair glucose homeostasis (Lambert and Lambert, 2011[66]; Eiden, 2013[29]). Kvetnansky et al., presented evidence illustrating that stress changes peripheral and circulating catecholamine levels via transcriptional and post-transcriptional pathways (Kvetnansky et al., 2013[65]). They reported that chronic stress caused a persistent rise in circulating norepinephrine and systemic blood pressure and a decreased level of catecholamine receptors in different regions of the nervous system, such as sympathetic ganglia, and in the medulla of the adrenal glands (Kvetnansky et al., 2013[65]). Bruce et al., provided evidence indicating that psychological stress impaired insulin sensitivity via ANS activation (Bruce et al., 1992[13]). Moreover, Licht et al., demonstrated that stress-induced ANS dysregulation could potentially predict insulin resistance. They found that stress-induced increases in levels of sympathetic activity impaired insulin sensitivity in patients under psychological stress, probably through modulation of circulating adipokines or catecholamines (Licht et al., 2013[69]). Moreover, Jarczok and coworkers in 2016 reported that work stress dysregulated glycemic control and induced insulin resistance via ANS hyperactivation (Jarczok et al., 2016[52]). 

The association between stress and ANS activity is complicated and not yet fully understood. However, the roles of transcriptional and post-transcriptional processes are prominent (Kvetnansky et al., 2013[65]). Prolonged stress is able to modulate catecholamine receptor expression in certain regions of the nervous system, such as sympathetic ganglia, in the medulla of the adrenal glands and in cardiac tissues (Kvetnansky et al., 2013[65]). Moreover, it can impact central nervous areas controlling ANS activities, such as nuclei in the cortex, hypothalamus and spinal cord (De Diego et al., 2008[24]). Chronic stress also has a clear impact on the adrenal glands, which are closely involved in ANS activities (Eiden, 2013[29]). Hence, chronic stress modulates ANS activities through different circuits (De Diego et al., 2008[24]). In total, these clinical data confirm that stress-induced insulin resistance is, in part, mediated by ANS overactivation.

### 5. Stress and renin-angiotensin system

As previously noted, the RAS is a hormonal system affecting most tissues by generating the active metabolite angiotensin II, which is a potent vasoconstrictor produced through sequential cleavage processes and has potent regulatory roles on hemodynamic/electrolyte homeostasis (Phillips et al., 2018[99]). In addition to fluid homeostasis, RAS is involved in insulin signaling and also in the pathophysiology of DM (Lim et al., 2004[71]; Ribeiro-Oliveira et al., 2008[104]; Simoes e Silva et al., 2017[117]). Previous observations suggest that RAS activation modulates glucose metabolism (Lim et al., 2004[71]; Ribeiro-Oliveira et al., 2008[104]; Simoes e Silva et al., 2017[117]). Angiotensin II is not only involved in diabetic microvascular complications, including retinopathy and nephropathy, but is also involved in insulin resistance (Ribeiro-Oliveira et al., 2008[104]). RAS activity interferes with the insulin signaling pathway at several points (Muscogiuri et al., 2008[90]; Ribeiro-Oliveira et al., 2008[104]; Zhou et al., 2012[146]). For example, angiotensin II reduces PI3K activity (Zhou et al., 2012[146]); it also attenuates Glut-4 localization on cell membranes of insulin sensitive tissues (Stump et al., 2006[121]; Zhou et al., 2012[146]). Angiotensin II is able to impair IST via a mitogen-activated protein kinase (MAPK) dependent mechanism (Steinberg et al., 1994[119]; Zhou et al., 2012[146]). Additionally, angiotensin II negatively impacts nitric oxide (NO) bioavailability which, in turn, impairs IST and reduces insulin sensitivity (Steinberg et al., 1994[119]). The RAS has extensive effects on the tissues involved in glucose metabolism, such as pancreas and liver (Simoes e Silva et al., 2017[117]). It can also stimulate pathophysiological pathways involved in insulin resistance, such as endoplasmic reticulum (ER) stress, oxidative stress, inflammation and islet fibrosis (Simoes e Silva et al., 2017[117]).

Correspondingly, RAS blockers and/or angiotensin converting enzyme (ACE) inhibitors are able to reverse these deleterious effects of angiotensin II by improving insulin sensitivity and pancreatic beta cell function and thus are promising therapeutic targets for DM (Lau et al., 2004[67]; Ribeiro-Oliveira et al., 2008[104]; Wei et al., 2008[134]; Madec et al., 2013[78]; Simoes e Silva et al., 2017[117]). Hayashi et al., in 2014 reported that Irbesartan, an angiotensin II receptor blocker, improved stress-induced insulin resistance by up-regulating IRS-1 and Glut-4 and reducing inflammatory responses in adipocytes of C57BL/6J mice (Hayashi et al., 2014[45]). Pavlatou et al., in 2008 reported that another angiotensin II receptor blocker, Candesartan, attenuated stress and anxiety dependent insulin resistance in patients with T2DM (Pavlatou et al., 2008[97]). They found that 3 months of Candesartan therapy attenuates the stress dependent CRH response and reduces circulating CRH levels in T2DM patients (Pavlatou et al., 2008[97]). Similarly, Gong and coworkers in 2019 found that Candesartan improved brain insulin signaling in stressed rats by promoting IRS-1 expression. They demonstrated that Candesartan improved behavioral changes as well as insulin sensitivity in these animals (Gong et al., 2019[39]). These findings support our hypothesis that stress-induced insulin resistance is mediated, at least partly, via RAS hyperactivation. 

### 6. Stress and antagonizing insulin action (endocrine abnormalities) 

The stress response is commonly associated with endocrine abnormalities such as high cortisol and low sex steroid levels (Yaribeygi and Sahraei, 2018[143]). These abnormalities can exert antagonizing effects on insulin and disrupt its hypoglycemic function (Björntorp, 1999[10]; Geer et al., 2014[36]). These effects may be compensated for in the acute phase; however, under chronic stress exposure, they can become a risk factor for developing insulin resistance as has previously been reported (Qi and Rodrigues, 2007[101]; Geer et al., 2014[36]; Zhou et al., 2016[147]). Elevated levels of glucocorticoids and reduced concentrations of sex hormones are predictors for insulin resistance (Qi and Rodrigues, 2007[101]; Geer et al., 2014[36]; Zhou et al., 2016[147]). Therefore, it is highly likely that endocrine abnormalities that commonly occur in response to stress provides another linkage between stress and insulin resistance (Qi and Rodrigues, 2007[101]; Geer et al., 2014[36]; Zhou et al., 2016[147]).

## Conclusion

DM and stress are mutually dependent. While DM is an upstream event for stress, it may also be an outcome of chronic stress. Epidemiological studies have confirmed that DM is a common stress-driven disease and stressful life events are positively correlated with a higher incidence of diabetes. Individuals who are regularly stressed are more prone to develop DM due to an unhealthy lifestyle, hyperphagia, lack of physical activity and a reduced tendency to use medications. Stress and insulin resistance are likely linked through molecular interactions (Table 2(Tab. 2); References in Table 2: Anichkov et al., 2005[7]; Bebbington et al., 2019[9]; Bruce et al., 1992[13]; Chuang et al., 2010[20]; Geer et al., 2014[36]; Gong et al., 2019[39]; Hayashi et al., 2014[45]; Himmerich et al., 2008[47]; Huffman et al., 2013[51]; Jarczok et al., 2016[52]; Johnson et al., 2017[53]; Lambert and Lambert, 2011[66]; Licht et al., 2013[69]; Lieberman et al., 2012[70]; Lyra e Silva et al., 2019[77]; Mancia et al., 2007[79]; Mellon et al., 2019[86]; Parekh et al., 2017[94]; Parkulo, 2014[95]; Pavlatou et al., 2008[97]; Postal et al., 2016[100]; Qi and Rodrigues, 2007[101]; Rodríguez-Sureda et al., 2007[105]; Saavedra, 2012[107]; Shiloah et al., 2003[114]; Simoes e Silva et al., 2017[117]; Suozzi et al., 2009[122]; Zhou et al., 2016[147]). In the present review, we introduce six possible molecular pathways, including pancreatic beta cells, lipid metabolism, the renin-angiotensin system, the autonomic nervous system, the immune response system and endocrine hormones which are under influence of stress. Despite growing evidence in clinical studies, more extensive investigations are still required. We suggest that future studies examine the effects of stress elements (such as different hormones, neurotransmitters and stress systems) on insulin sensitivity in both physiological (minor stress) and pathological states. 

## Notes

Habib Yaribeygi and Amirhossein Sahebkar (Biotechnology Research Center, Pharmaceutical Technology Institute, Mashhad University of Medical Sciences, Mashhad, Iran; E-mail: amir_saheb2000@yahoo.com) contributed equally as corresponding author.

## Declaration

### Funding

There is no funding in this study.

### Competing interests

The authors declare that there is no conflict of interest.

### Data availability

There is no raw data associated with this review article.

### Acknowledgment

The authors are thankful to the “Clinical Research and Development Unit" of the Baqiyatallah Hospital (Tehran, Iran)” for providing technical supports.

## Figures and Tables

**Table 1:**
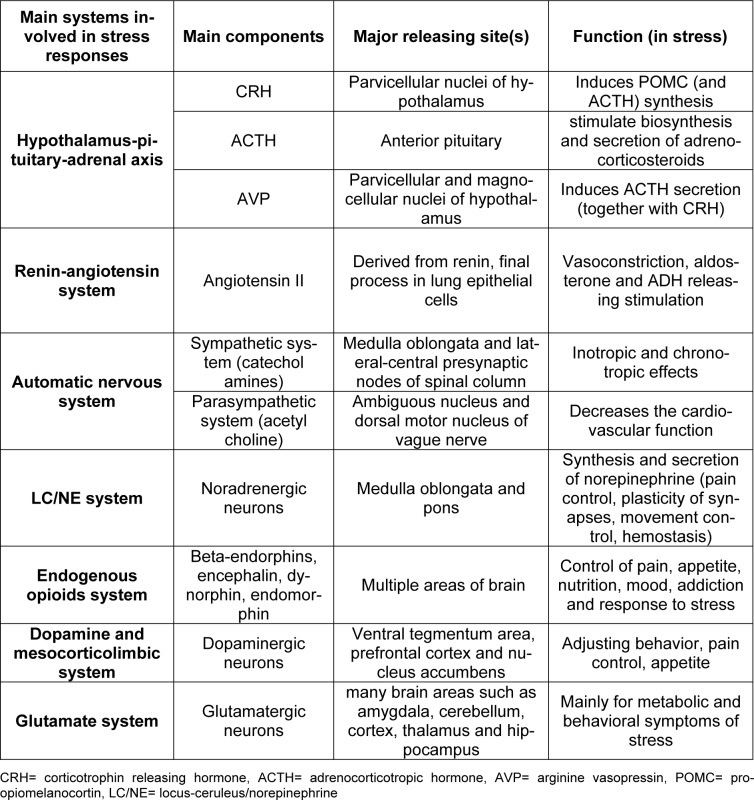
Main physiological systems, and their major components, that are involved in the stress response

**Table 2:**
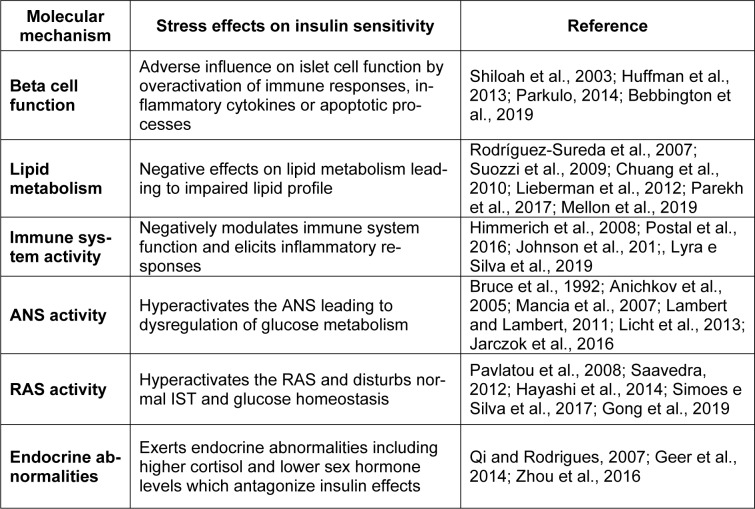
Possible molecular interactions between stress and insulin resistance (RAS=renin-angiotensin system, ANS=autonomic nervous system, IST=insulin signal transduction)
